# A Cross-Sectional Study Revealing the Emergence of Erythromycin-Resistant *Bordetella pertussis* Carrying *ptxP3* Alleles in China

**DOI:** 10.3389/fmicb.2022.901617

**Published:** 2022-07-18

**Authors:** Xiaoying Wu, Qianqian Du, Dongfang Li, Lin Yuan, Qinghong Meng, Zhou Fu, Hongmei Xu, Kaihu Yao, Ruiqiu Zhao

**Affiliations:** ^1^Department of Infectious Diseases, Children’s Hospital of Chongqing Medical University, National Clinical Research Center for Child Health and Disorders, Ministry of Education Key Laboratory of Child Development and Disorders, Chongqing Key Laboratory of Child Infection and Immunity, Chongqing, China; ^2^Beijing Key Laboratory of Pediatric Respiratory Infection Diseases, MOE Key Laboratory of Major Diseases in Children, Beijing Pediatric Research Institute, Beijing Children’s Hospital, Capital Medical University, National Center for Children’s Health, Beijing, China; ^3^BGI Pathogenesis Pharmaceutical Technology, BGI-Shenzhen, Shenzhen, China; ^4^Department of Respiratory Diseases, Children’s Hospital of Chongqing Medical University, National Clinical Research Center for Child Health and Disorders, Ministry of Education Key Laboratory of Child Development and Disorders, Chongqing Key Laboratory of Pediatrics, Chongqing, China

**Keywords:** *Bordetella pertussis*, virulence-related genotypes, erythromycin-resistance, genome sequence, *ptxp3*, molecular epidemiology

## Abstract

**Background:**

Previous limited studies have identified that *Bordetella pertussis (B. pertussis)* isolates circulating in China possess distinct molecular features and high rates of erythromycin-resistance (ER). Their evolution and potential impact on the prevention and control of global pertussis are worthy of attention.

**Methods:**

The present cross-sectional study involved 311 non-duplicate and unrelated *B. pertussis* strains isolated from Chinese children from 2017 to 2019. Their antimicrobial susceptibilities were assessed using both *E*-test strips and Kirby-Bauer (KB) disk diffusion methods. Seven virulence-related genes (*ptxA*, *ptxC*, *ptxP*, *prn*, *fim2*, *fim3*, and *tcfA2*) and the A2047G mutation in the 23S rRNA gene were detected by PCR. Based on the susceptibilities and genotypes, 50 isolates were selected for multi-locus variable-number tandem-repeat analysis (MLVA) typing and whole-genome sequencing.

**Results:**

A total of 311 *B. pertussis* strains were isolated from children with a median age of 4 months (interquartile range: 2–9 months). Strains carrying the *ptxP1* allele were more frequent (84.9%, 264/311), were always ER (except for one strain), and were mainly related to *ptxA1/ptxC1/prn1* alleles (99.6%, 263/264). The remaining 47 (15.1%) strains carried the *ptxP3* allele, mainly harboring the *ptxA1/ptxC2/prn2* alleles (93.6%, 44/47), and were sensitive to erythromycin (except for two strains). The two ER-*ptxP3* isolates were first identified in China, belonged to MT27 and MT28 according to MLVA, and were classified into sub-lineage IVd by phylogenetic analysis of their genome sequences. This sub-lineage also includes many strains carrying the *ptxP3* allele spreading in developed countries. For each tested antimicrobial, the susceptibilities judged by KB disks were consistent with those determined by *E*-test strips.

**Conclusion:**

The present results reveal that *B. pertussis* strains with the *ptxP1*-ER profile still dominate in China, and a few strains carrying the *ptxP3* allele have acquired the A2047G mutation in the 23S rRNA gene and the ER phenotype. The surveillance of the drug susceptibility of *B. pertussis* is necessary for all countries, and the KB disk method can be adopted as a screening test.

## Introduction

Pertussis, an acute pandemic and vaccine-preventable respiratory infection caused by *Bordetella pertussis* (*B. pertussis*), is an important cause of respiratory illness and death in children worldwide (Cherry, 2016; Mbayei et al., 2019; Skoff et al., 2019). Due to widespread inoculation with whole-cell pertussis vaccines (WCVs) beginning in the 1940s, pertussis cases have declined markedly (Güriş et al., 1999; Roush et al., 2007; Bart et al., 2014; Cherry, 2016), and disease mortality has declined by 78% globally (Gopal et al., 2019). However, since the 1990s, some developed countries with high pertussis vaccination coverage have experienced a pertussis resurgence coinciding with the period in which acellular pertussis vaccines (ACVs) replaced the WCVs, and more countries began reporting this phenomenon later. As such, the resurgence of pertussis has become a global health problem (Güriş et al., 1999; Bart et al., 2014; Lam et al., 2014; Consortium et al., 2019). In 2012, the United States reported >48,000 pertussis cases, the highest number since the 1950s (Skoff et al., 2019). Improved diagnostics, increased awareness, waning immunity, the shift from WCVs to less-effective ACVs, and antigen variations of the pathogen have been implicated as the key factors responsible for the increase in the number of cases (Bart et al., 2014).

Whole-cell pertussis vaccines were first administered in China in the early 1960s, but the country began switching to ACVs in 2007. After a short period of co-vaccination with WCVs and ACVs between 2007 and 2012, ACVs completely replaced WCVs in 2013 (Xu et al., 2015b). Two different techniques are used to produce ACVs: co-purification and separate purification. Separately purified ACVs (sp-ACVs) have been widely used worldwide, whereas co-purified ACVs (cp-ACVs) were adopted for national immunization programs in a few countries, including China (Xu et al., 2015b). Pertussis toxin and filamentous hemagglutinin are the main antigens contained in cp-ACVs, although trace amounts of other antigens, such as pertactin (Prn), may not be completely removed (Wang et al., 2012). In China, diphtheria-tetanus-WCVs and diphtheria-tetanus-ACV are administered at 3, 4, and 5 months of age for primary immunizations and 18–24 months of age for booster immunizations (Wang et al., 2012; Liu et al., 2019). An imported sp-ACV (Sanofi) has been available for infants and toddlers in China since 2011; however, its coverage is low because it is used in the private sector and mainly supplied in developed cities. During 2009–2018, the immunization coverage rate of the three primary doses of pertussis vaccine in children in China has been >99%. However, pertussis vaccination cannot be performed in school-aged children, adolescents, adults, and pregnant women in China.

In the past decade, the number of reported pertussis cases in China has increased significantly. The annual number was less than 3,000 between 2011 and 2013 (Ning et al., 2018), but began to increase sharply after that, with 3,408 cases in 2014, 6,658 in 2015, 5,584 in 2016, 10,390 in 2017, 22,057 in 2018, and 30,027 in 2019 (Ning et al., 2018; Yao et al., 2020). Researchers have speculated that the number of reported cases may grossly underestimate the actual scope of the epidemic in China due to insufficient surveillance capacity (Ning et al., 2018; Feng et al., 2021).

In many countries, the circulating *B. pertussis* strains have evolved to present primarily a non-vaccine antigen genotype (*ptxA1/prn2/ptxP3*) and exhibit good fitness to vaccine-induced selection pressure (Octavia et al., 2012; Bowden et al., 2014; Barkoff et al., 2018; Zomer et al., 2018; Carriquiriborde et al., 2019). Currently, *ptxP3* with/without *prn*-negative strains has expanded globally (Bowden et al., 2014; Lam et al., 2014; Barkoff et al., 2018; Zomer et al., 2018; Bouchez et al., 2021). One study collecting 256 isolates from nine European countries between 2012 and 2015 showed that the alleles *ptxA1*, *ptxP3*, and *prn2* account for 100, 95.5, and 96.2%, respectively, whereas 24.9% (66/265) of isolates exhibit *prn* deficiency (Barkoff et al., 2018). Although the macrolides have been the first-choice antibiotics to treat pertussis for more than 50 years worldwide, a few countries other than China (e.g., the United States, France, and Iran) have reported occasional erythromycin-resistant (ER) strains that do not exhibit epidemic trends (Bartkus et al., 2003; Guillot et al., 2012; Mirzaei et al., 2015). However, previous studies revealed the unique molecular characteristics and common ER phenotype of circulating *B. pertussis* strains in China (Fu et al., 2019; Li et al., 2019; Xu et al., 2019; Zhang et al., 2020). These studies discovered that the *ptxA1/ptxP1/prn1* genotype was dominant among bacterial isolates, with the proportion ranging from 53.3 to 97.6%, and the *ptxA1/ptxP3/prn2* genotype was relatively less common, ranging from 1.9 to 45.7% (Fu et al., 2019; Li et al., 2019; Xu et al., 2019; Zhang et al., 2020). Moreover, almost all *ptxP1* strains were determined to be ER, whereas the *ptxP3* strains were identified as erythromycin-susceptible (ES) without exception (Yang et al., 2015; Li et al., 2019).

Previous studies have revealed the widespread distribution of *ptxP1*-ER strains in China (Yao et al., 2020). Maybe due to the frequency of international travel in modern societies, researchers from Vietnam and Japan recently have reported pertussis cases involving infection with ER strains and demonstrated that these ER isolates had spread from Chinese isolates (Kamachi et al., 2020; Yamaguchi et al., 2020). The Global Pertussis Initiative indicated that the emergence of ER *B. pertussis* may pose a potentially looming threat to global public health and may further complicate the epidemiology of pertussis (Feng et al., 2021). Epidemic ER strains are worthy of attention. The present study was conducted as a continuation of a previous surveillance study of clinical *B. pertussis* strains aimed at elucidating the evolution of molecular characteristics and antimicrobial susceptibility of *B. pertussis* populations in mainland China.

## Materials and Methods

### Demographic Data and Clinical Information Regarding Bacterial Strains

This was a cross-sectional epidemiologic study conducted between January 2017 and December 2019. Nasopharyngeal specimens were collected from all children suspected of having pertussis at five tertiary hospitals in China, and the specimens were subjected to *B. pertussis* culture. In total, 311 *B. pertussis* strains were isolated from non-duplicate pediatric cases with no epidemiologic links to each other.

The geographic site of each case was considered the patient’s residential address, and age was calculated to the bacterial sampling date. The use of antibiotics before sample collection was verified in 157 cases, and the vaccination history was verified for 153 cases by checking the individual’s immunization records. Additionally, the medical records of 97 inpatients in Beijing Children’s Hospital and Children’s Hospital of Chongqing Medical University were available and reviewed for clinical analysis.

Informed, written consent was obtained from the parents or the legal guardians of each participant before samples were collected. The present study was approved by the Ethics Committee of each of the five hospitals: Children’s Hospital of Chongqing Medical University, Beijing Children’s Hospital Affiliated with Capital Medical University, Jiaxing University Affiliated Women and Children’s Hospital, Wuhu No. 1 Hospital/Wuhu Children’s Medical Center, and Nanjing Children’s Hospital. There were no ethical concerns pertaining to the study.

### Identification of *Bordetella pertussis*

Nasopharyngeal specimens were promptly inoculated onto charcoal agar (OXOID, United Kingdom) plates containing 10% defibrinated sheep blood and *Bordetella* selective supplement (OXOID, United Kingdom). The plates were incubated in a humidified incubator at 35–37°C for 3–5 days. Any suspected colonies were tested via slide agglutination using *B. pertussis* and *B. parapertussis* antiserum (Remel Europe Ltd., United Kingdom). Confirmed *B. pertussis* strains were obtained in pure cultures and then stored at −80°C.

### Antimicrobial Susceptibility Testing

The susceptibility of each of the 311 isolates to erythromycin, ampicillin, levofloxacin, gentamycin, and sulfamethoxazole/trimethoprim (SXT) was determined simultaneously using *E*-test strips (bio-Merieux, SA, France) and KB disks (Oxoid Ltd., Basingstoke, United Kingdom) on charcoal agar medium without selective supplementation. As there were not enough SXT *E*-test strips for all isolates, the minimum inhibitory concentration (MIC) of SXT was determined for 234 isolates. The KB disks used for this study contained 15 μg of erythromycin, 10 μg of ampicillin, 10 μg of gentamycin, 5 μg of levofloxacin, or 25 μg of SXT. The tests were performed as previously reported (Li et al., 2019). The MICs and inhibition zone diameters were read after 4 days of incubation and adopted for the analysis. *Staphylococcus aureus* ATCC 29213 and *Haemophilus influenzae* ATCC49247 were used for quality control in the tests. According to previous studies, ES strains were defined as exhibiting a MIC ≤ 0.5 mg/L and/or an inhibition zone diameter ≥42 mm (Li et al., 2019; Cimolai, 2021a). The breakpoints of *H. influenzae* against SXT, ampicillin, and levofloxacin and the breakpoint of *Escherichia coli* against gentamycin recommended by the Clinical and Laboratory Standards Institute (Clinical Laboratory Standards Institute [CLSI], 2019) were utilized as references for determining susceptibility in this study.

### Genomic DNA Extraction and Gene Sequencing

The strains were cultured at 35–37°C for 72 h and then sub-cultivated for an additional 72 h on fresh charcoal agar plates containing 10% defibrinated sheep blood without *Bordetella* selective supplementation. Genomic DNA was extracted using a DNA extraction kit (Tiangen Biotech Co., Ltd, Beijing, China) according to the manufacturer’s instructions.

Seven virulence-related genes (*ptxA*, *ptxC*, *ptxP*, *prn*, *fim2*, *fim3*, and *tcfA2*) and the 23S rRNA gene of *B. pertussis* were amplified and sequenced following procedures published previously (Yang et al., 2015; Mooi et al., 2000). The mutation A2047G in domain V of the 23S rRNA gene was responsible for ER (Bartkus et al., 2003; Guillot et al., 2012; Mirzaei et al., 2015; Xu et al., 2019), and genotypes were determined as described previously (Li et al., 2019).

### Multi-Locus Variable–Number Tandem-Repeat Analysis

A total of 50 strains (Supplementary Table 1) were selected for multi-locus variable–number tandem-repeat analysis (MLVA) and genome sequencing. Seven isolates with rare phenotypes or gene alleles were initially selected, including two *ptxP3*-ER strains, one non-ER *ptxP1* strain, and four strains harboring rare *prn* or *fim3* alleles. Next, 43 isolates were randomly selected. The final 50 selected strains included 28 *ptxP1* strains and 22 *ptxP3* strains. By the combination of six variable–number tandem repeats (VNTR1, VNTR3a, VNTR3b, VNTR4, VNTR5, and VNTR6), the MLVA types were verified according to previously described procedures (Li et al., 2019; Schouls et al., 2004). Based on the six MLVA loci, a minimum spanning tree was developed using BioNumerics, version 7.6 (Applied Maths^1^).

### Whole-Genome Sequencing, Single-Nucleotide Polymorphism Identification, and Phylogenetic Analysis

Whole-genome sequencing and sequence analysis of the 50 isolates (Supplementary Table 1) were performed as previously described (Yao et al., 2020). Briefly, paired-end reads of 100 bp for each strain were generated on an Illumina HiSeq X platform according to standard protocols. On average, 626 Mbp of sequencing data were obtained for each strain, with a depth of 152× (Supplementary Table 1). To identify variations in the present strains, some of the sequence data for 98 strains among five distinguished lineages from our previous study were included in the present analysis (Yao et al., 2020). The whole-genome sequence of strain Tohama I (NC_002929, Japan) was used as the mapping reference genome. The 3,193 high-quality single-nucleotide polymorphisms (SNPs) identified in our previous study were adopted to construct a phylogenetic tree using iq-tree (version: 1.6.11) based on the maximum-likelihood method (Yao et al., 2020). In order to evaluate the homology of the present lineage IVd covering the two *ptxP3*-ER strains with epidemic strains in other countries (e.g., Europe, Australia, and North American countries), another phylogenetic tree was constructed for the present sequences in the IVd lineage and other published sequences in lineage IV (Supplementary Table 2) (Bart et al., 2014; Theofiles et al., 2014; Xu et al., 2015a; Yao et al., 2020; three available datasets through NCBI: PRJNA356412, PRJEB21744, PRJNA432286). The two *ptxP3*-ER strains were further sequenced to determine the complete genome sequence (using PacBio sequencing).

### Statistical Analyses

Statistical analyses were performed using SPSS 25.0 (IBM, Chicago, IL, United States). The χ*^2^* test, modified χ*^2^* test, Fisher’s exact test, Student’s *t*-test, and Mann–Whitney *U*-test were conducted, as appropriate. Non-normally distributed data are presented as median (interquartile range, IQR), and numerical data are presented as percent (%). A two-sided *P*-value < 0.05 was considered statistically significant.

## Results

### Demographic Diversity

**FIGURE 1:**
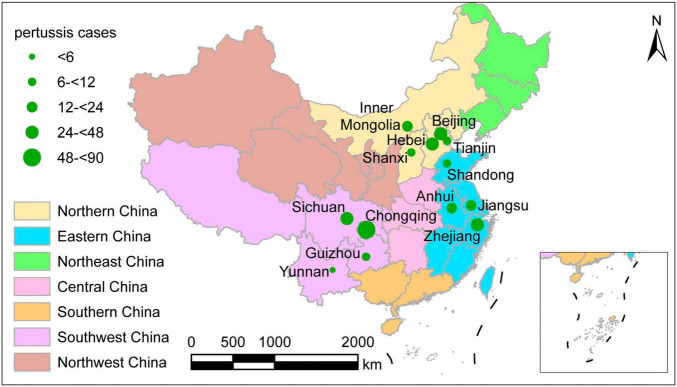
Geographic distribution of the 311 culture-confirmed pertussis cases at the time of onset, mainland China, 2017–2019. Different colors represent different geographic regions. The size of green circles indicates the number of patients in the province.

The geographic distribution of the 311 culture-confirmed pertussis cases is shown in Figure 1, covering three of the seven geographic areas of mainland China, including 13 provinces, municipalities, and autonomous regions. One hundred of the patients lived in the southwest part of China and included 61 cases in Chongqing, 25 in Sichuan, 9 in Guizhou, and 5 in Yunnan. Ninety-three cases lived in eastern China, including 48 in Zhejiang, 21 in Anhui, 17 in Jiangsu, and 7 in Shandong. The remaining 118 cases lived in northern China and included 44 patients in Beijing, 40 in Hebei, 8 in Shanxi, 12 in Tianjin, and 14 in the Inner Mongolia Autonomous Region. The distribution of the 311 isolates by year was 64 (20.5%) in 2017, 128 (41.2%) in 2018, and 119 (38.3%) in 2019. The patients included 165 men and 146 women. The median age of all cases was 4 months (interquartile range: 2–9 months). Nine neonates were diagnosed with pertussis, and an 84-month-old boy was the oldest patient among these cases. The age distribution of the patients included 88 (28.3%) younger than 3 months, 108 (34.7%) aged 3–5 months, 81 (26.1%) aged 6–17 months, and 34 (10.9%) older than 18 months.

### Antimicrobial Susceptibility and 23S rRNA Gene Variations

The results of antimicrobial susceptibility tests are shown in Table 1. A total of 265 strains (85.2%) exhibited a MIC > 256 mg/L against erythromycin and a diameter of 6 mm in KB tests (no inhibition zone around the disks). The A2047G mutation in the 23S rRNA gene was identified in these 265 isolates. The remaining 46 (14.8%) isolates exhibited MICs ranging between 0.094 and 0.25 mg/L to erythromycin according to *E*-test strips, and the inhibition zone diameter ranged from 43 to 54 mm in KB disk tests. No A2047G mutation in the 23S rRNA gene was identified in these 46 isolates. In addition, no heterogeneous resistance phenotype was observed. The MIC values against SXT, ampicillin, levofloxacin, and gentamicin were always low, and the inhibition zone diameter was >18 mm. With regard to the breakpoints of *H. influenzae* and *E. coli*, all isolates were susceptible to these four antimicrobials.

**TABLE 1:** Antimicrobial susceptibility of the presented 311 *Bordetella pertussis* isolates in China during 2017–2019.

	Antimicrobial	*E*-test (mg/L)				Kirby–Bauer (KB) disk diffusion (mm)		
			
		MIC_50_^a^	MIC_90_^b^	MIC range	*S*^c^(*n*)	Median	Range	*S* (*n*)
Total, *n* = 311	Erythromycin	>256	>256	0.094–>256	46	6	6–54	46
	Ampicillin	0.5	0.75	0.19–1	311	39	32–44	311
	Levofloxacin	0.38	0.5	0.19–0.75	311	27	19–32	311
	Gentamycin	1	1.5	0.25–2	311	29	25–34	311
	SXT*^d^*	0.125	0.38	0.008–0.5	234	30	18–43	311
*ptxP1*, *n* = 264	Erythromycin	>256	>256	0.125–>256	1	6	6–48	1
	Ampicillin	0.5	0.75	0.19–1	264	39	32–44	264
	Levofloxacin	0.38	0.5	0.19–0.75	264	27	19–32	264
	Gentamycin	1	1.5	0.25–2	264	29	25–34	264
	SXT	0.125	0.38	0.008–0.5	193	30	18–43	264
*ptxP3*, *n* = 47	Erythromycin	0.125	0.25	0.094–>256	45	49	6–54	45
	Ampicillin	0.5	0.75	0.19–1	47	41	33–44	47
	Levofloxacin	0.38	0.5	0.25–0.75	47	28	22–31	47
	Gentamycin	1	1.5	0.75–1.5	47	29	26–32	47
	SXT	0.19	0.38	0.064–0.5	41	28	19–36	47

*^a^MIC_50_: 50th percentile of MIC values.*

*^b^MIC_90_: 90th percentile of MIC values.*

*^c^S: susceptible.*

*^d^SXT: sulfamethoxazole/trimethoprim, as there were not enough SXT E-test strips for all isolates, and the minimum inhibitory concentration (MIC) of SXT was determined for 234 isolates.*

### Comparison Between Clinical Characteristics of Pertussis Cases Caused by Erythromycin-Resistant and Erythromycin-Susceptible Isolates

Compared with patients with pertussis caused by ES strains, cases caused by ER strains were more frequently prescribed macrolides (primarily erythromycin and azithromycin) and/or β-lactams (primarily cephalosporin and amoxicillin) until the presented samples were taken for bacterial cultures (Supplementary Table 3). The ER group had a longer course of paroxysmal cough, and more cases were complicated with pneumonia compared with the ES group (Table 2).

**TABLE 2:** Clinical characteristics of the pertussis cases caused by erythromycin-resistant (ER) strains and cases caused by erythromycin-sensitive (ES) strains in China, 2017–2019.

	Total *n* = 97	ER patients *n* = 83	ES patients *n* = 14	*P*-value
*ptxP3*, *n* (%)	14(14.4)	0(0)	14(100)	NA^a^
Younger than 3 months old, *n* (%)	40(41.2)	32(38.6)	8(57.1)	0.19
Pertussis vaccination, *n* (%)	46(47.4)	40(48.2)	6(42.9)	0.71
Living in countryside, *n* (%)	28(28.9)	26(31.3)	2(14.3)	0.33
Disease duration at antimicrobial initiation(d)(IQR)^b^	7(5–10)	7(4.75–10)	6(4.5–9)	0.38
Disease duration at admission(d)(IQR)	15(11–20)	18(14–20)	11(8.5–12.5)	<0.001
Tachypnea, *n* (%)	26(26.8)	20(24.1)	6(42.9)	0.25
Cyanosis, *n* (%)	49(50.5)	44(53)	5(35.7)	0.23
Asphyxia or apnea, *n* (%)	4(4.1)	3(3.6)	1(7.1)	0.47
Complication of pneumonia, *n* (%)	68(70.1)	62(74.7)	6(42.9)	0.04
Complication of severe pneumonia, *n* (%)	6(6.2)	5(6.0)	1(7.1)	1
Extrapulmonary organs damage*^c^*, *n* (%)	34(35.1)	29(34.9)	5(35.7)	1
Peak WBC count (×10^9^/L) (IQR)	18.9(14.1–27.1)	19.4(14.2–28.0)	17.1(12.4–20.7)	0.33
Peak WBC count >20 × 10^9^/L, *n*(%)	45(46.4)	40(48.2)	5(35.7)	0.39
Co-infection with other pathogens, *n* (%)	39(40.2)	34(41.0)	5(35.7)	0.71
Special therapies*^d^*, *n* (%)	25(25.8)	23(27.7)	2(14.3)	0.46
ICU admission, *n* (%)	3(3.1)	3(3.6)	0	NA
Survival, *n* (%)	97(100)	83(100)	14(100)	NA
Disease duration at paroxysmal cough remission(d)(IQR)	23(18–29)	24(20–29)	18(15–19.75)	0.002
Length of hospital stay(d)(IQR)	7(5–10)	7(6–10)	7.5(4.75–12)	0.84

*^a^not applicable.*

*^b^IQR: interquartile range; information was available in 74 out of 83 patients caused by ER strains and 13 out of 14 patients caused by ES strains.*

*^c^Extrapulmonary organs involved in liver, heart, or brain here.*

*^d^Special therapies included administering the treatment of glucocorticoids, IVIG, exchange transfusion, or mechanical ventilation here.*

### Virulence-Related Genotypes

Except for one strain of the *fim3-2* type, all strains exhibited the common *ptxA1/fim3-1/fim2-1/tcfA2* profile. Different profiles were determined based on three genes: *ptxC*, *ptxP*, and *prn*. The two most common profiles were *ptxC1/ptxP1/prn1* (84.5%, 263/311) and *ptxC2/ptxP3/prn2* (14.5%, 45/311).

In total, six profiles for the seven virulence-related genes were identified (Table 3).

**TABLE 3:** Distribution by year and geographic region of the seven virulence-related genotypes among the presented 311 *B. pertussis* isolates in China, 2017–2019.

	Total [*n* (%)]	Year Distribution [*n* (%)]			Geographic Distribution [*n* (%)]		
			
		2017	2018	2019	South west	Eastern China	Northern China
PtxA1/ptxC1/ptxP1/prn1/fim3-1/fim2-1/tcfA2	263(84.5)	58(91)	107(83.6)	98(77.3)	87(87.0)	66(71)	110(93.2)
PtxA1/ptxC2/ptxP3/prn2/fim3-1/fim2-1/tcfA2	44(14.2)	6(10)	20(15.6)	18(15.2)	12(12.0)	25(27)	7(5.9)
PtxA1/ptxC2/ptxP3/prn3/fim3-1/fim2-1/tcfA2	1(0.3)	0	0	1(0.8)	1(1.0)	0	0
PtxA1/ptxC2/ptxP3/prn9/fim3-1/fim2-1/tcfA2	1(0.3)	0	0	1(0.8)	0	1(1)	0
PtxA1/ptxC1/ptxP1/prn12/fim3-1/fim2-1/tcfA2	1(0.3)	0	0	1(0.8)	0	1(1)	0
PtxA1/ptxC2/ptxP3/prn2/fim3-2/fim2-1/tcfA2	1(0.3)	0	1(0.8)	0	0	0	1(0.9)
Total	311	64	128	119	100	93	118

The geographic distribution showed that the *ptxP3* type was more common among strains isolated from eastern China (26/93, 28%) than among strains isolated from the northern region (8/118, 6.8%; χ*^2^* = 17.26, *P* < 0.001) or southwest region (13/100, 13.0%; χ*^2^* = 6.69, *P* = 0.01).

**FIGURE 2:**
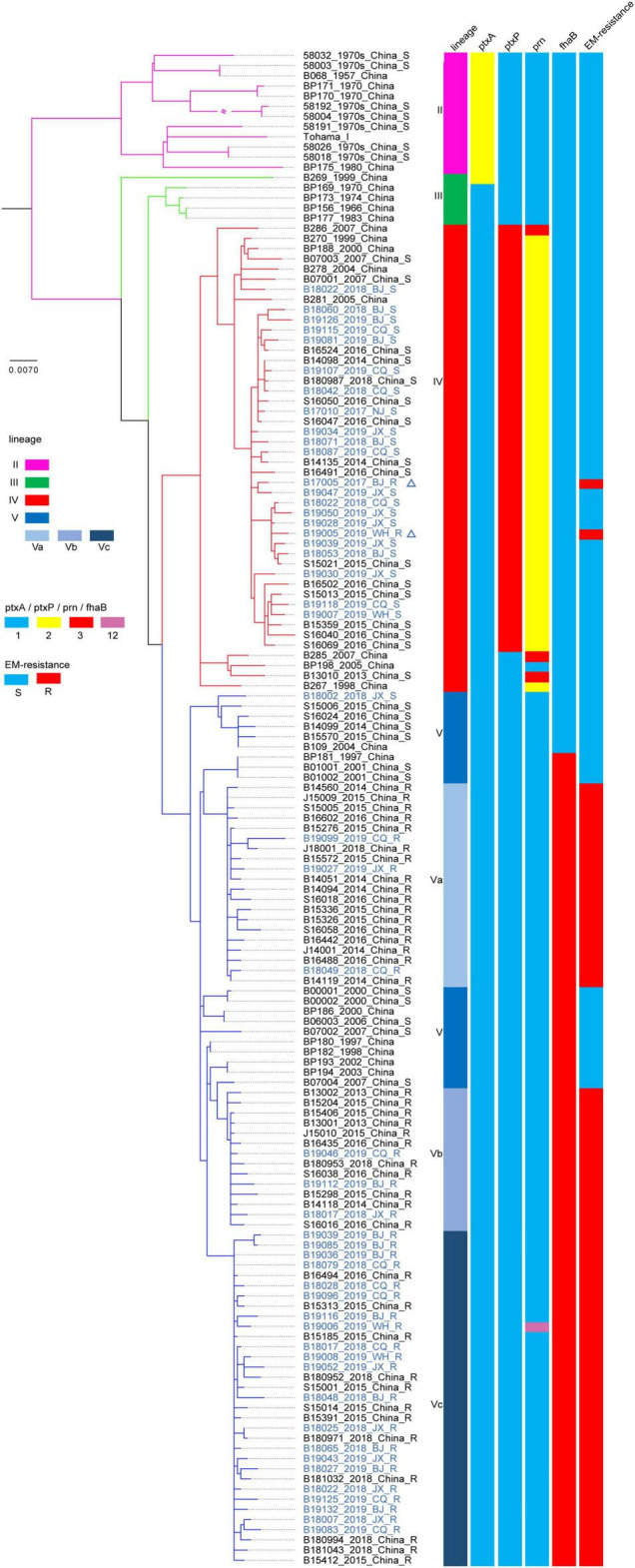
Maximum clade credibility phylogenetic tree for Chinese *Bordetella pertussis* isolates in the present and previous studies based on whole-genome single-nucleotide polymorphism (SNPs). Maximum clade credibility phylogenetic tree for 148 Chinese *B. pertussis* isolates. The leaves are marked as “sample ID_ isolated year_isolated area_erythromycin resistance.” The leaves representing the strains in the present study are colored blue. The two leaves marked by triangles represent the location of two *ptxP3*-ER (erythromycin-resistance) strains in the present study. Vertical bars on the right display the lineage, *ptxA*, *ptxP*, *prn*, *fhaB* allele type, and ER for each strain or sub-lineage. The legend is on the left side of the figure.

Almost all strains harboring the *ptxP1* gene (263/264) showed the ER phenotype. Surprisingly, 2 (B17005_2017_BJ_R and B19005_2019_WH_R) of the 47 strains identified as the *ptxP3* type also exhibited an ER phenotype (Figure 2), which was further confirmed by complete genome sequencing for the *ptxP3* genotype and A2047G mutation in the 23S rRNA gene. Strain B17005_2017_BJ_R was isolated from a patient living in Jiangsu Province in 2017, and strain B19005_2019_WH_R was isolated from a patient living in Anhui Province in 2019 (Supplementary Figure 1).

### Multi-Locus Variable–Number Tandem-Repeat Analysis

**FIGURE 3:**
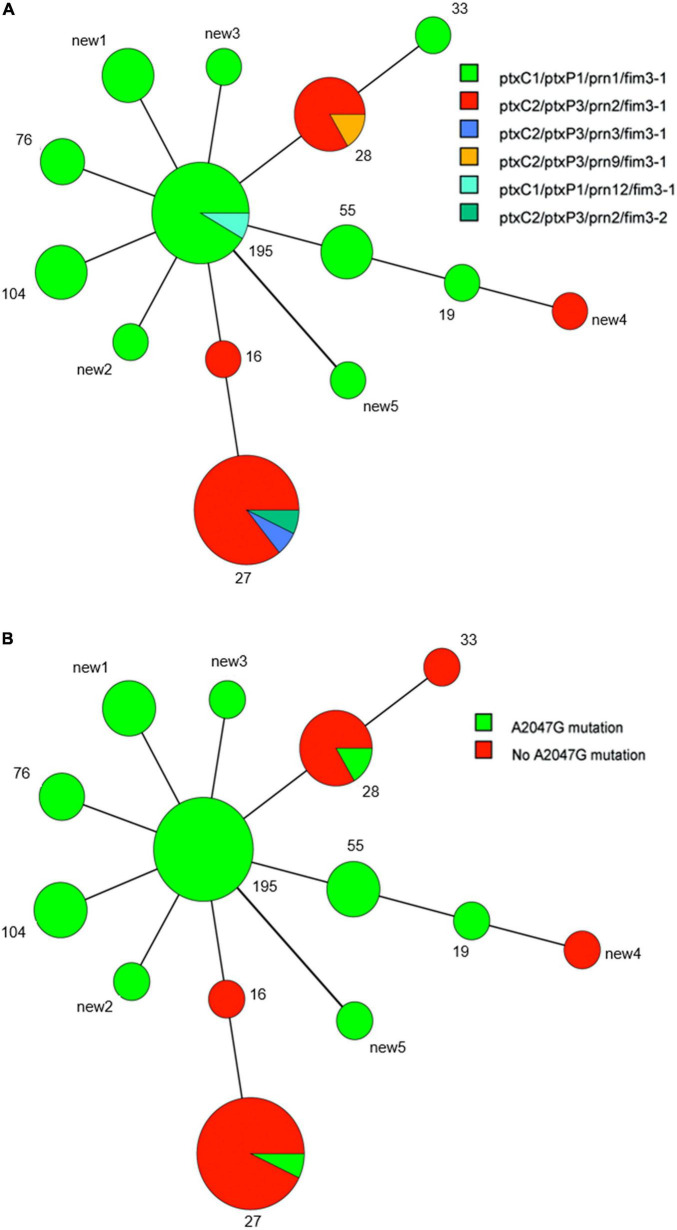
Minimum spanning tree of multi-locus variable-number tandem-repeat analysis (MLVA) types of 50 *B. pertussis* isolates in the present study. Each circle indicates an MLVA type, and the type number is next to the circle. Circle size indicates the number of isolates categorized according to the particular MLVA type. Differences in the length and thickness of the lines linking two circles indicate differences in the number of VNTRs between the two linked MLVA types. Different colors of circles denote different allelic patterns of virulence antigen genes and different erythromycin susceptibilities. **(A)** Allelic patterns. **(B)** Presence or absence of the A2047G mutation.

Fourteen MLVA patterns were found among the 50 tested isolates, which included MT27 (14 isolates), MT195 (12 isolates), MT28 (6 isolates), MT55 (5 isolates), MT104 (3 isolates), MT76 (2 isolates), MT16 (1 isolate), MT19 (1 isolate), MT33 (1 isolate), and five new types named new1 through new5. Each of the five new MTs was represented by a single isolate, except for new1, which was represented by three isolates. The six VNTRs (VNTR1, VNTR3a, VNTR3b, VNTR4, VNTR5, and VNTR6) of the five newly identified MLVA profiles were 8-6-0-8-6-8 in new1, 9-6-0-7-6-8 in new2, 8-6-0-7-5-8 in new3, 8-6-7-7-6-5 in new4, and 8-5-7-7-6-8 in new5. MT195 (8-6-0-7-6-8) was the predominant MT type in previous Chinese reports. New1, new2, and new3 were different single-locus variants of MT195, whereas new4 and new5 were double-locus variants of MT195. Only MT28 and MT27 covered both ES and ER isolates, with the two *ptxP3*-ER isolates representing the two ER ones (Figure 3).

### Whole-Genome Sequencing

**FIGURE 4:**
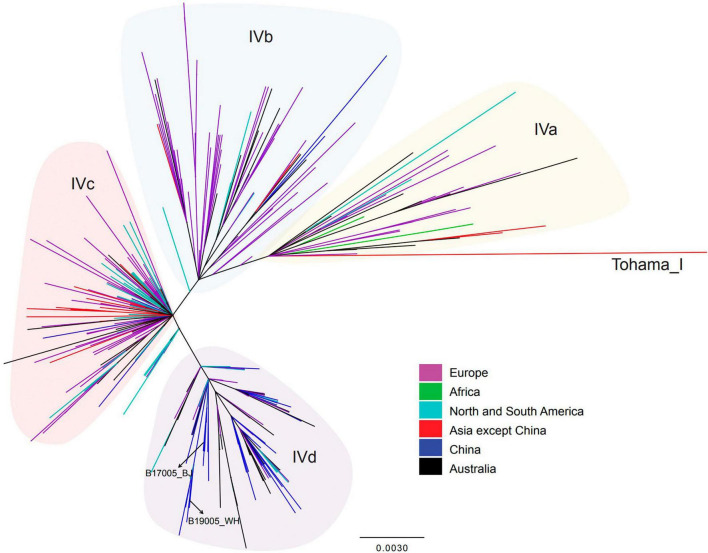
Single-nucleotide polymorphisms-based phylogenetic tree for lineage IV *B. pertussis* strains isolated from China and other countries. Maximum-likelihood phylogenetic tree of 358 *B. pertussis* isolates from China (8 isolates from the present study and 46 from the previous study) and other countries (304 isolates), based on whole-genome SNPs. According to the legend, the branch colors indicate the isolate resource (country). Shadow colors indicate lineages. Basic information regarding the strains included in this figure is presented in Supplementary Table 2.

The phylogenetic tree constructed based on SNPs exhibited five lineages, as previously determined (Figure 2). The 50 isolates sequenced in the present study were defined only into lineage IV or V. The 28 isolates harboring the *ptxP1* gene belonged to lineage V, primarily sub-lineage Vc. The remaining 22 isolates harboring the *ptxP3* gene belonged to lineage IV, and most were concentrated in sub-lineage IVd. In particular, the two *ptxP3*-ER strains also belonged to lineage IVd. Further analysis revealed a close genetic relationship between the strains in lineage IV, irrespective of the country or region in which the strains were isolated (Figure 4). A comparison between the complete genome sequences of the two *ptxP3*-ER isolates revealed 12 non-synonymous base substitutions, 4 synonymous substitutions, and 4 intergenic substitutions. In contrast to strain B19005_2019_WH_R and all *ptxP1*-ER strains, strain B17005_2017_BJ_R harbored the A2047G mutation in only two of the three copies of the 23S rRNA gene, which exhibited two peaks at the 2047 point in the PCR amplification (Supplementary Figure 2). Analysis of the genomes of the 50 isolates showed that the *fhaB3* allele was harbored by all *ptxP1*-ER isolates, and the *fhaB1* allele was harbored by all *ptxP3* isolates and the single *ptxP1*-ES isolate. In addition, we did not detect any *prn* gene deletion or insertion sequences in any of the 50 strains.

The whole-genome sequences and complete genome sequences were deposited in the NCBI Sequence Read Archive, BioProject: PRJNA770762 and PRJNA769544, respectively.

## Discussion

Previous studies have shown that *B. pertussis* isolates circulating in mainland China are typically *ptxP1*-ER strains. The present results revealed that *ptxP1*-ER strains continue to dominate the *B. pertussis* population in China, whereas most *ptxP3* strains retain sensitivity to erythromycin. The two present *ptxP3*-ER strains were the first such confirmed ER *ptxP3* isolates in China.

The present *ptxP1*-ER epidemic is similar to previous reports in China (Fu et al., 2019; Li et al., 2019; Xu et al., 2019), in contrast to the situation in many developed countries (Bowden et al., 2014; Barkoff et al., 2018). The overuse of macrolides in China is a key factor determining the prevalence of China-specific *ptxP1*-ER isolates (Yao et al., 2020). The high rate of antibiotic administration and predominance of ER strains in positive cultures are suggestive of the failure of erythromycin treatment in China, as recently demonstrated in clinical research by Mi et al. (2021). The present cases of pertussis caused by *ptxP1* strains seemed more severe than those caused by *ptxP3* strains. This is contrary to what has been observed in other countries (Mooi et al., 2009). It is well-known that timely treatment with antibiotics to which isolates are sensitive can diminish the severity of pertussis (Cimolai, 2021b). The opposite finding in terms of disease severity, therefore, could be related to the fact that early treatment with macrolides can eliminate *ptxP3*-ES strains but not *ptxP1*-ER strains.

It has been confirmed that the ER isolates are also resistant to other macrolides (Li et al., 2019). In contrast, the present results showed the *B. pertussis* strains were significantly inhibited by SXT, which is recommended for pertussis therapy and prophylaxis. Ampicillin has been shown to strongly inhibit *B. pertussis in vitro*, but its clinical efficacy is debated (Li et al., 2019; Cimolai, 2021b). Mi et al. (2021) recently reported that β-lactam antibiotic treatment is more effective for the clearance of ER *B. pertussis* than macrolide treatment. The tested isolates were commonly sensitive to levofloxacin and gentamycin. However, little clinical evidence is currently available, indicating that pertussis treatment or prophylaxis with quinolones or aminoglycosides is efficacious (Cimolai, 2021b). Notably, several of the present isolates sensitive to SXT and ampicillin exhibited higher MICs (SXT 0.5 mg/L; ampicillin 1 mg/L) in the present tests. Several *B. pertussis* isolates exhibiting MICs as high as 32 mg/L for SXT (indicating resistance) have appeared in Zhejiang Province in China (Hua et al., 2019). Therefore, future surveys should focus on the antimicrobial susceptibility of the *B. pertussis* strains not only to macrolides but also to alternative non-macrolide agents.

The widespread emergence of ER *B. pertussis* strains prompted the urgent necessity of finding an appropriate method for pertussis susceptibility testing. Without standardized methods and recommended breakpoints worldwide, most studies have used ***E***-test strips to accurately determine the MICs of antimicrobials against *B. pertussis* (Cimolai, 2021a). An accurate, simple, reproducible, and cost-effective method is needed for the clinical selection of antimicrobial agents and epidemiologic surveys. This and previous studies have reported high MICs of erythromycin (32 to >256 mg/L) for resistant strains (Yang et al., 2015; Korgenski and Daly, 1997), which differed markedly from the MICs for sensitive strains (≤ 0.5 mg/L) (Cimolai, 2021a). Such a sharp contrast between resistant and sensitive strains was also demonstrated by KB disk tests, which revealed two distinct result patterns in the present study: 6 mm (no inhibition zones around the disks) vs 43–54 mm. Almost all of the ES strains exhibited a diameter ≥ 43 mm for erythromycin disks, except for one strain that exhibited a 36-mm zone in previous reports (Li et al., 2019; Yang et al., 2015). Based on the above data, an obvious inhibition zone in a KB test could signify the isolate is sensitive to erythromycin. Furthermore, the present results found that the susceptibilities to the five tested agents as judged by the KB method agreed with the ***E***-test results. Both the ***E***-test and KB disk diffusion test can be adopted for susceptibility testing in drug susceptibility surveys. The latter has an advantage in terms of cost-effectiveness and ease of operation.

The occurrence of *ptxP3*-ER strains in China is likely due to strong ER selection and the local prevalence of *ptxP3* strains in individual regions (Zhang et al., 2020). In contrast to previous investigations in China that found ER is always linked to *B. pertussis* strains harboring the *ptxP1* type and that *ptxP3* strains are ES without exception (Li et al., 2019; Yao et al., 2020), the present tests identified the first two *ptxP3*-ER strains in China, which belonged to lineage IV. Lineage IV is reportedly always sensitive to erythromycin (Yao et al., 2020). One *ptxP3*-ER *B. pertussis* strain was reported by Guillot et al. (2012) in France. However, the spread of such strains in Europe or other countries could not be traced. Because the genome of the French strain was not available, its association with the present *ptxP3*-ER strains could not be evaluated. However, considering the long time interval between the two events and the resistance only in *ptxP1* type strains in China during 2012–2019 (Feng et al., 2021), the occurrence of the present two *ptxP3*-ER strains appears to represent a new variation event without a link to the French strain. Furthermore, the two *ptxP3* strains likely acquired the resistance mutation separately, because they were isolated in different provinces and years. More importantly, they had different copies of the A2047G mutation and MLVA types. However, both strains could have evolved from a common ancestor (Figures 2, 4).

China primarily uses a two-component cp-ACV targeting Pertussis toxin and filamentous hemagglutinin. The CS strain (Chinese vaccine strain) harbors *ptxA2-ptxP1-prn1-fhaB1* alleles. Perhaps as a result of the selection pressure from vaccination, our study indicated that the *fhaB3* allele (estimated to have emerged in the early 1990s) has expanded in nearly all *ptxP1* strains but not *ptxP3* strains, consistent with the results of another Chinese study (Xu et al., 2019). With more antigenic variations (Li et al., 2019), the *ptxP3* strain likely holds a competitive advantage over the *ptxP1* strain under vaccine-driven selection pressure and has therefore been a global epidemic agent despite the various immunization strategies employed in different countries and regions (Octavia et al., 2012; Bowden et al., 2014; Barkoff et al., 2018; Zomer et al., 2018; Carriquiriborde et al., 2019). These strains should theoretically be prevalent in China as well; however, the overuse of macrolides in China could limit the spread of *ptxP3*-ES strains and promote the spread of epidemic *ptxP1*-ER strains (Yao et al., 2020). The identification of *ptxP3*-ER strains in the present investigation may affect the macrolide selection limitation of their spread not only in China but also in other countries. More importantly, the international spread of the *ptxP3-prn2* (Yao et al., 2020), MT27 (Barkoff et al., 2018; Li et al., 2019; Xu et al., 2019), MT28 (Barkoff et al., 2018; Mir-Cros et al., 2019), and lineage IV (Yao et al., 2020) types has demonstrated that *ptxP3* strains have a strong capacity to adapt to immune selection pressure. Therefore, the emergence of *ptxP3*-ER strains may pose a potentially difficult challenge for pertussis prevention and control worldwide if they could spread to a critical degree in China. However, aside from the differences in the *ptxP* and *prn* alleles, the Chinese *ptxP1*-ER strains and global *ptxP3* strains also differ in terms of the *fhaB* allele (*fhaB3* vs *fhaB1*) (Xu et al., 2019). Therefore, global *ptxP3-fhaB1* strains exhibiting macrolide resistance may not survive better than *ptxP1-fhaB3*-ER strains in China, because the Chinese vaccine strain CS harbors the *fhaB1* gene (Xu et al., 2019), and immune selective pressure on *fhaB1* strains is greater. Further investigations will be needed to monitor the actual spread of *ptxP3*-ER strains and determine their influence in China and abroad.

The emergence of Prn-deficient strains may be the result of immune selection pressure exerted by sp-ACVs containing a Prn component. The longer the period since the introduction of an ACV containing Prn, the greater the potential for a higher frequency of circulating Prn-deficient isolates in a country (Barkoff et al., 2019). Currently, the frequency of such Prn-deficient strains is low in China because Prn is not an effective antigen in the cp-ACVs. To date, only one Prn-deficient strain has been suspected in China (Xu et al., 2019). Genomic analyses of China isolates both in our previous study and the present sequencing study have not found any *prn* gene deletions or insertions (Yao et al., 2020).

This study has several limitations. First, the *B. pertussis* isolates were collected from limited (three of seven) geographic regions of mainland China. However, the present collection in the southwest region of China made up for the deficiency in previous studies and provides additional evidence on the nationwide spread of ER *B. pertussis* in China. Second, this study was not based on patient selection but rather on positive *B. pertussis* cultures. The high rate (93.6%) of antibiotic use before sampling in this study undoubtedly decreased the rate of positive *ptxP3*-ES cultures. Thus, the prevalence of *ptxP3* strains in China may have been underestimated. In addition, the high rate of antibiotic use before sampling may have led to the underestimation of the prevalence of *ptxP1*-ES isolates too. Third, the number of patients in the ER and ES groups could not be adequately matched. This may have affected the accuracy of statistical analyses regarding clinical characteristics between the two groups. In the future, more cases should be examined to better study the clinical features of pertussis caused by ER *B. pertussis* strains.

In summary, the results of the present study reveal that *B. pertussis* strains with the *ptxP1*-ER profile are still dominant in China and that a few strains carrying the *ptxP3* allele have acquired the A2047G mutation in the 23S rRNA gene and consequently the ER phenotype. The *ptxP3*-ER strains have appeared sporadically in China, and these strains could pose a potential challenge for pertussis prevention and control efforts if they spread more widely. Continuous monitoring of the genetic changes and antimicrobial susceptibility of *B. pertussis* strains is needed in order to improve pertussis prevention and treatment strategies and to provide scientific data for the development of new vaccines. The KB disk diffusion test can be adopted to screen the antimicrobial susceptibility of *B. pertussis* strains because of its accuracy, cost-effectiveness, and ease of operation.

## Data Availability Statement

The datasets presented in this study can be found in online repositories. The names of the repository/repositories and accession number(s) can be found in the article/Supplementary Material.

## Ethics Statement

The studies involving human participants were reviewed and approved by The Ethics Committees of Children’s Hospital of Chongqing Medical University, Beijing Children’s Hospital Affiliated to Capital Medical University, Jiaxing University Affiliated Women and Children Hospital, Wuhu No. 1 Hospital/Wuhu Children’s Medical Center, and Nanjing Children’s Hospital. Written informed consent to participate in this study was provided by the participants’ legal guardian/next of kin.

## Author Contributions

XW, QD, LY, RZ, HX, QM, KY, and ZF collected the isolates in their local sites. KY and RZ applied the funds to support this work. XW, KY, and RZ designed the study and interpreted the results. XW, QD, LY, and KY conducted the experiments. DL was responsible for the whole-genome and complete-genome sequencing and analysis work with the assistance of XW, QD, QM, RZ, ZF, and KY. XW and QD collected the data and performed statistics. XW, QD, RZ, HX, ZF, and KY drew the pictures and made the tables. XW wrote the first draft of the manuscript. QD, HX, and ZF wrote parts of the manuscript. KY and RZ revised the manuscript according to all authors’ comments before submission and responsible for the manuscript. All authors contributed to revisions of the manuscript, read, and approved the submitted version.

## Conflict of Interest

DL was employed by BGI PathoGenesis Pharmaceutical Technology, BGI-Shenzhen. The remaining authors declare that the research was conducted in the absence of any commercial or financial relationships that could be construed as a potential conflict of interest.

## Publisher’s Note

All claims expressed in this article are solely those of the authors and do not necessarily represent those of their affiliated organizations, or those of the publisher, the editors and the reviewers. Any product that may be evaluated in this article, or claim that may be made by its manufacturer, is not guaranteed or endorsed by the publisher.

## Footnotes

1
https://www.applied-maths.com
