# Supplementary material for: A Cross-Sectional Study Revealing the Emergence of Erythromycin-Resistant Bordetella pertussis Carrying ptxP3 Alleles in China
Source: Front Microbiol. 2022 Jul 18;13:901617. doi: 10.3389/fmicb.2022.901617 (PMC9342848; doi:10.3389/fmicb.2022.901617)
Supplement: Supplementary file 1 [file Data_Sheet_1.PDF]

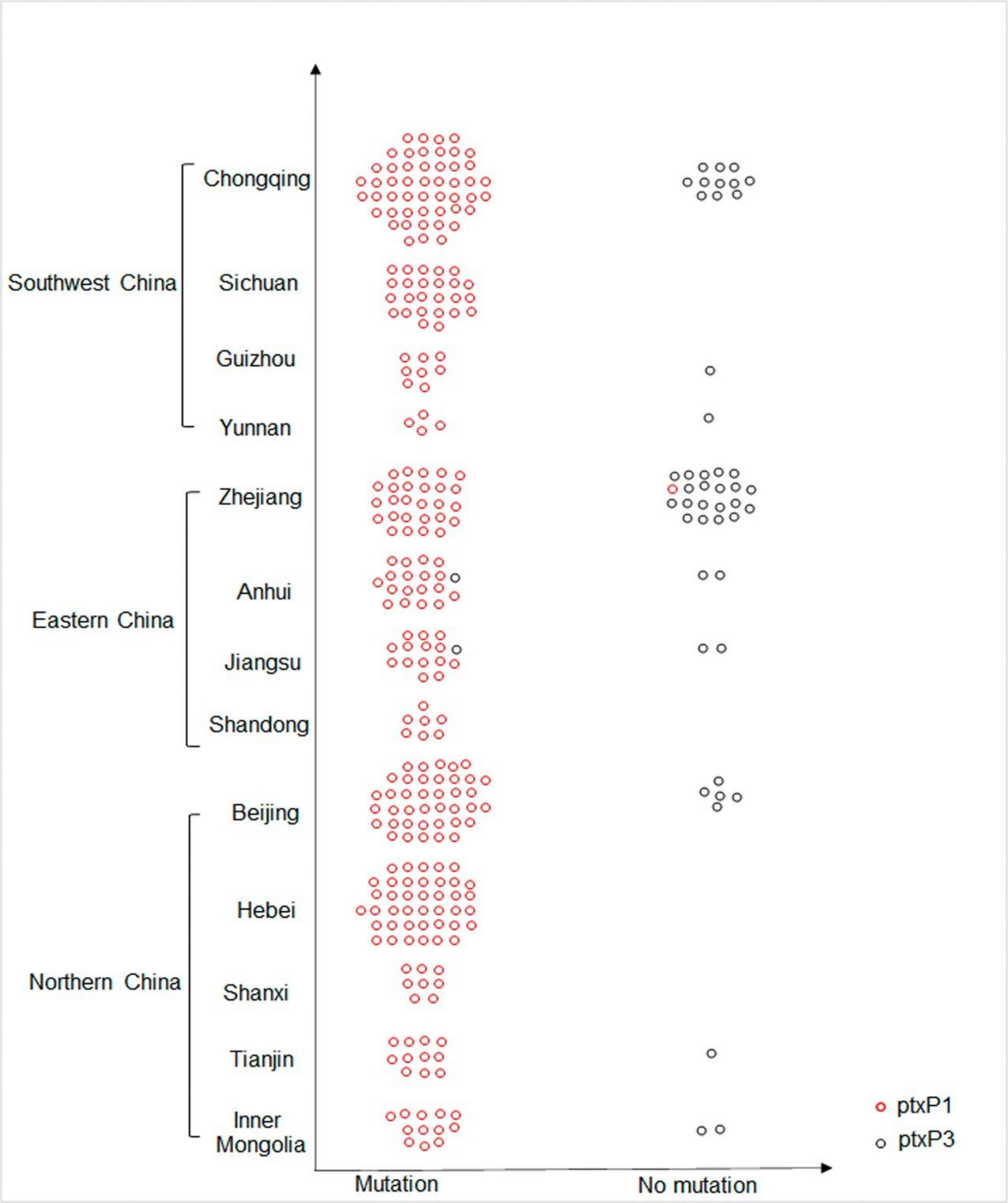

Supplementary figure 1. Geographic distribution of *ptxP* alleles and erythromycin resistance of 311 *Bordetella pertussis* isolates in mainland China, 2017-2019.

Panel A

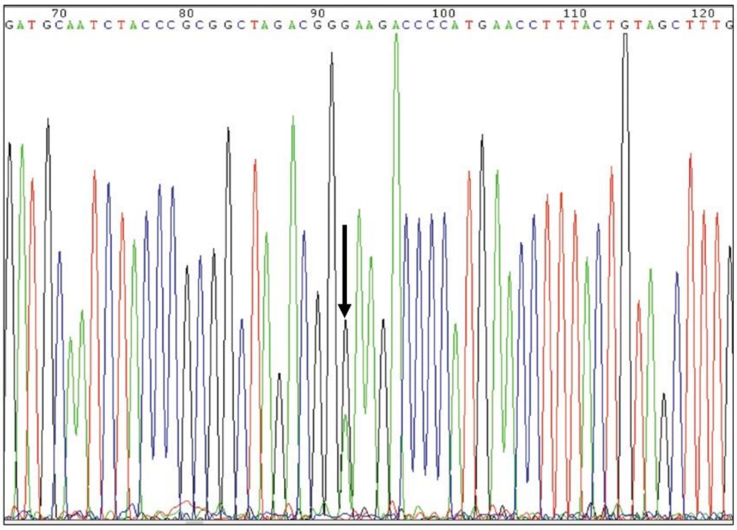

Panel B

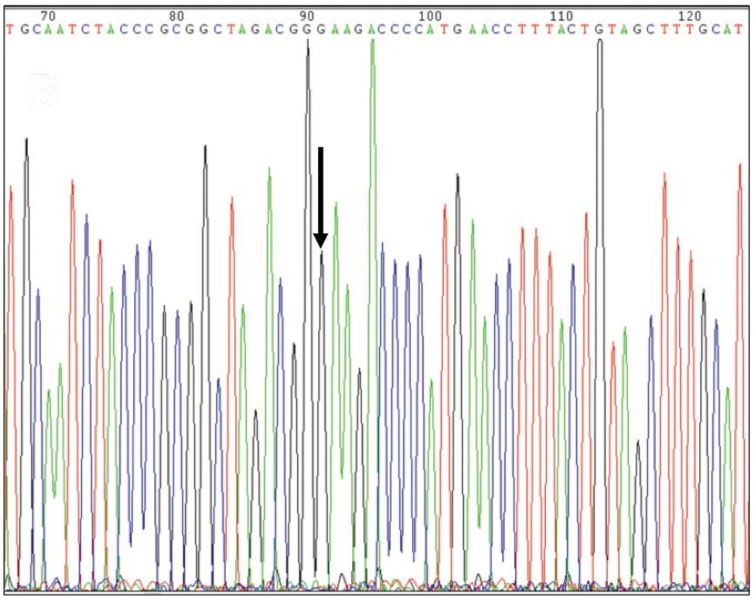

Supplementary figure 2. The sequencing peak map of A2047G mutation in 23S rRNA for two *ptxP3*-erythromycin resistance *Bordetella pertussis* isolates in mainland China, 2017-2019.

Panel A: The black arrow represents the A2047G mutation peak in B17005_2017_BJ_R, which has the mutation in two of the three copies of 23S rRNA gene and shows two crests at the 2047 point.

Panel B: The black arrow represents the A2047G mutation peak in B19005_2019_WH_R, which has the mutation in all the three copies of 23S rRNA gene and shows one crest at the 2047 point.
