# Supplementary material for: A Cross-Sectional Study Revealing the Emergence of Erythromycin-Resistant Bordetella pertussis Carrying ptxP3 Alleles in China
Source: Front Microbiol. 2022 Jul 18;13:901617. doi: 10.3389/fmicb.2022.901617 (PMC9342848; doi:10.3389/fmicb.2022.901617)
Supplement: Supplementary file 7 [file Table_6.DOCX]

**Supplementary table 3** Comparison of antimicrobials therapy before *Bordetella pertussis* culture between the pertussis cases caused by erythromycin **-**resistant (ER) strains and the cases caused by erythromycin **-**sensitive (ES) strains, in mainland China, 2017-2019.

|  | **Total**  ***n*=157** | **ER patients**  ***n*=126** | **ES patients *n*=31** | ***P* value** |
| --- | --- | --- | --- | --- |
| **Culture within 2 weeks of illness course, *n* (%)** | 74(47.1) | 48(38.1) | 26(83.9） | ＜0.001 |
| **Antimicrobials therapy before culture, *n* (%)** | 147(93.6) | 121(96) | 26(83.9） | 0.04 |
| **Duration of total antimicrobials therapy before culture(d)** | 7(4,11) | 8(4.5,11.5) | 3(2.75, 5.25） | ＜0.001 |
| **Macrolides^a^ therapy before culture, *n* (%)** | 118(75.2) | 105(83.3) | 13（41.9） | ＜0.001 |
| **Duration of macrolides therapy before culture, (d)** | 4(3,7) | 4(3,7) | 2(1,3.5) | ＜0.001 |
| **β-lactam antimicrobials^b^ therapy before culture, *n* (%)** | 119(75.8) | 97(77.0) | 21（67.7） | 0.29 |
| **Duration of β-lactam antimicrobials therapy before culture(d)** | 4(2,7) | 5(2,7) | 3(1.5,4) | ＜0.001 |
| **Cases received both macrolides and β-lactam antimicrobials before culture, *n* (%)** | 89(56.7) | 81(64.3%) | 8(25.8) | ＜0.001 |
| **Pertussis vaccination^c^, *n* (%)** | 74(48.4) | 59(48.0) | 15(50.0) | 0.84 |

^a^ Here, macrolides primarily contain erythromycin and azithromycin.

^b^ Here, β-lactam antimicrobials primarily contain cephalosporins and amoxicillin.

^c^Vaccination history was collected in 153 out of 157 patients.
